# Building the case for mitochondrial transplantation as an anti-aging cardiovascular therapy

**DOI:** 10.3389/fcvm.2023.1141124

**Published:** 2023-05-09

**Authors:** Colwyn A. Headley, Philip S. Tsao

**Affiliations:** Stanford Cardiovascular Institute, Stanford University School of Medicine, Stanford, CA, United States

**Keywords:** aging, mitochondrial stress, ROS, oxidative stress, cardiovascular system, endothelial cells, vascular smooth muscle cells

## Abstract

Mitochondrial dysfunction is a common denominator in both biological aging and cardiovascular disease (CVD) pathology. Understanding the protagonist role of mitochondria in the respective and independent progressions of CVD and biological aging will unravel the synergistic relationship between biological aging and CVD. Moreover, the successful development and implementation of therapies that can simultaneously benefit mitochondria of multiple cell types, will be transformational in curtailing pathologies and mortality in the elderly, including CVD. Several works have compared the status of mitochondria in vascular endothelial cells (ECs) and vascular smooth muscle cells (VSMCs) in CVD dependent context. However, fewer studies have cataloged the aging-associated changes in vascular mitochondria, independent of CVD. This mini review will focus on the present evidence related to mitochondrial dysfunction in vascular aging independent of CVD. Additionally, we discuss the feasibility of restoring mitochondrial function in the aged cardiovascular system through mitochondrial transfer.

## Cardiovascular disease and aging

Cardiovascular disease (CVD) is a term that encompasses pathologies of the heart and blood vessels (1). Heart failure, congenital heart disease, coronary heart disease, peripheral artery disease, cerebrovascular disease, atherosclerosis, and abdominal aortic aneurysm (AAA) are all forms of CVD (1). Combined, CVD are the leading cause of death nationally and globally (2). For reference, in 2020 ∼32% of the global death burden was CVD-related (2). CVD etiology and pathophysiology are complex and mediated by several concurrent genetic and non-genetic risk factors (3). For example, risk factors such as hypertension, smoking, obesity, and aging contribute to the independent development of atherosclerosis and AAA (4). Individuals affected by diabetes mellitus type 2 are at a higher risk of developing atherosclerosis but are less likely to develop AAA (5). Yet, an individual with atherosclerosis may still develop AAA (5). Our current understanding of CVD pathophysiology is also confounded because several vascular cells (endothelial cells, vascular smooth muscle cells) and immune cells (macrophages and T cells) innervate multiple CVD (6).

Chronic inflammation, oxidative stress, and mitochondrial dysfunction (mito-dysfunction) are some of the conserved cellular dysregulations present in various CVD (7). Mechanistically, mito-dysfunction is directly linked to chronic inflammation and oxidative stress that plague the cardiovascular and immune systems (8–22). For example, mito-dysfunction in immune cells contributes toward increased inflammatory T cell polarization (20, 21), and irreversible M1 macrophage polarization (13). In the vasculature, mito-dysfunction contributes to endothelial cell (EC) dysfunction by decreasing the production of nitric oxide (NO), a potent vasodilator (8–12). Vascular smooth muscle cell (VSMC) phenotype and extracellular matrix (ECM) composition are also negatively impacted by mito-dysfunction (12, 18, 19). Mito-dysfunction-associated ATP depletion and increased mitoROS in cardiomyocytes are also well characterized in heart failure (14–17).

On the molecular level, several critical cellular processes succumb to aged-graded dysregulation (15, 23). These dysregulated processes, collectively known as the cellular hallmarks of aging, are also interconnected by oxidative stress and mito-dysfunction (15, 23). In the context of aging, mito-dysfunction is cell indiscriminate, and the magnitude of the mito-dysfunction present in an individual's immune and vascular cells can aggravate underlying CVD pathology (10, 11, 16, 22, 24–27). However, whether mito-dysfunction is primarily a consequence or instigator of CVD is still unresolved (24). Understanding the protagonist role of mitochondria in the respective and independent progressions of CVD and biological aging will unravel the synergistic relationship between biological aging and CVD. Moreover, the successful development and implementation of therapies that can simultaneously benefit mitochondria of multiple cell types, will be transformational in curtailing pathologies and mortality in the elderly (10, 22, 26). The impact of aging on cardiomyocyte mitochondria have been described in detail (17). Several works have also compared the status of mitochondria in vascular ECs and VSMCs in a CVD-dependent context (11, 16, 28). Herein we focus on the present evidence related to mitochondrial dysfunction in vascular aging independent of CVD. We, additionally discuss the feasibility of restoring mitochondrial function in the aged cardiovascular system through mitochondrial transfer.

## Structure of mitochondria

Structurally, a mitochondrion has 4 major partitions; the outer mitochondrial membrane (OMM), the inner mitochondrial membrane (IMM), an intermembrane space (IMS) that separates the OMM and IMM, and the mitochondrial matrix (29, 30). The OMM is primarily comprised of phospholipids, and interfaces with cytosolic organelles such as the endoplasmic reticulum, liposomes, peroxisomes, and the cytoskeleton (29, 30). While small uncharged molecules and ions can transverse freely across the OMM through porins (29, 30), larger proteins and molecules require the activity of translocases (TOMMS) (29, 30). The IMM folds within the mitochondrial matrix to create the mitochondrial cristae (29, 30). The transport of ions and molecules across the IMM into the mitochondrial matrix requires specific membrane transport proteins (TIMMS) (29, 30). Importantly, the mitochondrial matrix and the IMM are the respective sites of the tricarboxylic acid (TCA) cycle and oxidative phosphorylation (Ox-phos) (29, 30).

Ox-phos refers to the activity of the electron transport chain (ETC). The transfer of electrons across the 4 redox centers generates a proton gradient within the IMS, that subsequently powers the mitochondrial ATP synthase machinery. The electrons that enter the ETC originate from the reduced forms of NADH & FADH_2_. The tricarboxylic acid (TCA) is responsible for generating reduced NADH & FADH_2_. However, the TCA cycle is limited by the availability of Acetyl-CoA, which is derived from the catabolism of translocated pyruvate, fatty acids, and proteins (29–31). For these reasons, the stoichiometric relationships among mitochondrial Acetyl-CoA, TCA cycle intermediates, NADH, FADH_2_, O_2_, and ADP principally influence the rate of ATP derived from Ox-Phos (29–31). Consequently, mitochondria with compact cristae (i.e., densely structured IMM) outperform mitochondria with loose cristae in Ox-phos (substrate utilization and ATP generation) (32).

The redox chemistry of ETC, principally from complexes I & III, are major sources of mitoROS (33, 34). Several antioxidants such as SOD1-2, CAT, and GPxs are scattered within the mitochondrial matrix and counterbalance mitoROS accumulation (33–35). Accordingly, mitochondria architecture (size, shape, and volume) is important for optimizing the use of substrates related to mitochondrial respiration (36–39) and minimizing the mitoROS-induced-cellular toxicity (33–35). Further, beyond their role in ATP production, mitochondria participate in broad cellular signaling cascades including calcium, redox, and apoptotic signaling (40–42). Minute changes to the harmony of mitochondrial function can have a domino effect on other cellular processes (40).

## What is mito-dysfunction?

The term mito-dysfunction describes the acute or chronic inability of mitochondria to maintain optimal metabolic stoichiometry, and or mitochondrial architecture. Because mitochondrial processes are interconnected, determining whether singular or multiple incipient events are the root cause mito-dysfunction is challenging (43). Cells affected by aging-associated mito-dysfunction undergo concurrent cellular events that continually catalyze mito-dysfunction and cell dysregulations (44). Specifically, excess mitoROS production, abnormal assembly (biogenesis) and recycling (mitophagy) of mitochondria (i.e., abnormal mitochondrial mass), changes in mitochondrial DNA (mtDNA) quality and quantity, and shifts in mitochondrial respiration-related substrates are some of the prominent molecular readouts seen in aged cardiovascular cells (7, 12, 14, 24, 25, 45–47).

A decline in mtDNA copy number in peripheral leukocytes is associated with increased CVD risk and mortality (48, 49). Independent of CVD, limited studies have examined the aging-associated changes mitochondria undergo in vascular cells (16). Work by Jendrach et al. (47) and Ungvari et al. (12) provide some of the earliest quantifiable aging-associated changes to mitochondria. Jendrach et al. showed that compared to actively dividing primary human umbilical vein ECs (HUVEC) (young HUVEC), serially passaged/non-dividing (old) HUVEC contained swollen mitochondria with unstructured cristae, produced higher amounts of mitoROS, and harbored higher amounts of fragmented mtDNA (47). Ungvari et al. reported that vascular ECs from carotid arteries of 24-month-old rats have lower mitochondrial mass, lower proteomic expression of ETC complexes (I, III & IV), and increased mitoROS production, than vascular ECs of the carotid arteries from 3-month-old (young) rats (12). Similar results were also reported for VSMCs from carotid arteries of old rats (12). Work by Vendrov et al. reported increased mitoROS in aortic VSMCs from middle-aged mice (16 months) compared to young (4 months) (46). The mitochondria from aortic VSMCs from old mice also contained higher oxidized (carbonyl) residues, had decreased ETC activity (Complex I & III), and decreased mitochondrial respiration (oxygen consumption) (46).

The work by Ungvari et al. additionally reported that the mRNA levels of the mitochondrial transcription factor A (TFAM) and Peroxisome proliferator-activated receptor gamma coactivator 1-alpha (PGC1-α), another transcriptional coactivator of mitochondrial biogenesis, were decreased in the aortic tissue isolated from old rats, compared to young (12). Work by Foote et al. would later corroborate the aged-graded decrease in TFAM and PGC1-α expression, albeit in C57BL/6 mice (45). Foote et al. also reported an age-graded decline in mtDNA copy number in aortic tissue isolated from 8-, 22-, 44- and 72-week-old C57BL/6 mice (45). The metabolic activity (oxygen consumption rate; OCR) of mitochondria from aortic tissue also showed a concomitant age-graded decline (45). Separate work by Tyrrell et al. also reported a significant decline in the OCR of aortic mitochondria from old mice (18–19 months) when compared to young mice (2–3 months). Tyrrell et al. reported no difference in the levels of mtDNA damage (mtDNA breaks) between young and old C57BL6 mice (25). Interestingly, the aortic tissue of old mice contained significantly more mitophagy-associated proteins and ubiquitinated mitochondria, suggesting that mitophagy was upregulated in the aortic tissue of aged mice (25). Subsequent work by Tyrrell et al. later showed that under increasing mitochondrial stress, vascular tissue from aged mice do not have the capacity to concomitantly increase mitophagy, and this is opposite to what was seen in vascular tissue from young mice (50). These data ultimately point toward aging-associated impairment of mitophagy in vascular tissue (50).

## Mitochondrial DNA (mtDNA) damage accumulation

The quality and quantity of mtDNA, and its inherent location within cells are central to the heterogeneity of mito-dysfunction (51, 52). Mitochondria assembly is coordinated through the transcription and translation of nuclear (nDNA) and mtDNA-encoded genes, at a ratio of 99:1 (34, 52, 53). The quantitative contributions of mtDNA are far less than nDNA, however, mtDNA-encoded components are indispensable in the assembly of mitochondria. The size of mtDNA is also significantly smaller than nDNA (∼16 kbp vs. ∼3,300,000 kbp respectively) and mtDNA contains significantly fewer intron (non-coding DNA) regions (52, 54). Additionally, deletions and mutations in mtDNA often result in severe developmental defects and early life mortality (55).

Because mtDNA is located within the mitochondrial matrix, its DNA strands are susceptible to direct oxidation by mitoROS (56–59). Specific mitoROS, hydroxyl (OH•), and peroxyl (ROO•) radicals attack the nitrogenous bases and ribose backbones of mtDNA, creating oxidative lesions (56–59). In addition to mitochondrially localized antioxidants, TFAM also shields mtDNA from radical damage through non-specific binding (60). MtDNA is normally found as a compact nucleoid wrapped by TFAM (53). Alluding to its name, TFAM can also interact with mtDNA at specific binding domains to influence mtDNA synthesis/transcription and repair (53). TFAM has also been shown to bind more tightly to oxidized mtDNA residues (8-oxo-guanine), which likely is involved in promoting mtDNA repair (61). In the event of severe damage to an mtDNA nucleoid, mitochondria do contain multiple mtDNA nucleoids, allowing for the compensatory translation of non-defective proteins and tRNAs (55). DNA polymerase γ (Pol γ) is responsible for mtDNA replication and repair (62). However, Pol γ repairs oxidative-mtDNA lesions through base excision (BER), which may introduce base pair transversions and point mutations (63).

Additionally, intramitochondrial components, molecules, and enzymes, including Pol γ, are also highly susceptible to mitoROS-induced damage, which impacts their functionality. Regarding Pol γ, Anderson et al. showed that its proofreading and repair abilities are severely hindered under increasing levels of oxidative stress (62). Decreased Pol γ fidelity may further introduce point mutations and deletions to replicated mtDNA (64, 65). Adding to this, studies suggest that mtDNA with deletions are favorably replicated due to their size (66–68). In essence, depending on the burden of damage present within mitochondria, DNA polymerase γ can either facilitate the retention of healthy mtDNA or further aggrandize mito-dysfunction (69). Importantly, regardless of the cause of mtDNA damage, damaged mtDNA that escapes from the mitochondria into the cytosol can activate inflammatory responses, via TLR9, cGas-STING, and NLRP3 inflammasome signaling (70, 71). Chronic activation of these cellular responses contributes to the sterile inflammation seen in aging (70).

## Modulating mito-dysfunction

The augmentation of mitoROS dynamics has been one of the most effective inhibitory strategies in ameliorating mito-dysfunction *in vitro* and *in vivo* (72). However, decreasing mitoROS via modulating antioxidant defenses, reprogramming cellular metabolism, and preserving mtDNA quality have shown varied translational successes (73). Due to the conserved and complex role of redox signaling in cellular homeostasis, optimization of cell-specific dosing hinders the anti-aging therapeutic potential of antioxidants (73). Our current understanding of cellular reprogramming is still naïve, and the potential impact of *in vivo* de-differentiation toward oncogene activation needs to be further clarified (74, 75).

Several studies have also assessed whether directly modulating mtDNA is a viable therapeutic strategy (45, 76). The seminal *in vitro* cybrid work of Porteous et al. and colleagues exemplified how the thresholds of mtDNA heteroplasmy can manifest mito-dysfunction (77). The deletion in mtDNA^4799^, one of the most common mtDNA mutations detected in human diseases and associated with biological aging, impacts the expression of tRNAs, and several subunits of the ETC (I, IV, and V) (78). Porteous et al. created cybrids from mitochondrially depleted osteosarcoma cells and denucleated skin fibroblast from patients harboring mtDNA^4799^. Mitochondrial abnormalities including abnormal morphology, membrane potential, and ATP synthesis only manifested in cybrids harboring a mtDNA^4799^ heteroplasmy burden greater than 50% (77).

Regarding *in vivo* mtDNA heteroplasmy manipulation, most studies have been conducted in transgenic mice overexpressing nuclear-encoded genes, that relate to mtDNA stability and replication, particularly TFAM and the mtDNA helicase, Twinkle (51, 76, 79–81). Work by Ikeuchi et al. reported that the transgenic overexpression of human TFAM (hTFAM) in murine cardiomyocytes protected mice from myocardial ischemia-induced cardiac dysfunction and cardiac tissue remodeling (81). Interestingly, hTFAM is incapable of initializing murine mtDNA synthesis (80). Ikeuchi et al. postulated that the protective effect of hTFAM might be related to its ability to non-specifically bind murine mtDNA, thereby offering increased mtDNA stability, and prevented the decline in cardiac tissue Ox-Phos that normally follows heart failure (81). Additionally, hTFAM may have averted mito-dysfunction by shielding mtDNA from mitoROS. This reasoning is supported by the work of Xu et al., which showed that the overexpression of TFAM lacking the C-terminal tail, that is required for mtDNA transcription, still protected neuroblastoma mtDNA against oxidative damage (60).

A comparable study using hTFAM mice, by Ikeda et al., recapitulated and extended the observations originally made by Ikeuchi et al. (81). Additionally, Ikeda et al. examined whether Twinkle overexpression was cardioprotective during heart failure (76). The Twinkle overexpression was done using a murine construct (80). Consequently, when compared against non-transgenic wild-type controls, mtDNA synthesis was upregulated in the cardiac tissue of Twinkle mice (80). The genetic dissimilarities between hTFAM and Twinkle mice are obvious, yet when compared to wild-type mice, the cardiomyocytes isolated from both transgenic strains contained higher mtDNA copy numbers and lower steady-state levels of mitoROS (76). Moreover, devoid of the induction of heart failure, there were no significant differences in the ETC proteome, and ETC complex activities in hTFAM and Twinkle mice vs. wild type (76). Ikeuchi et al. noted no significant differences in the enzymatic activity of ETC complexes from hTFAM and WT mice (81), and Ikeda et al. reported no significant differences in the expression of mitochondrial antioxidant enzymes (76).

Unfortunately, these studies do not provide closure regarding the full cardioprotective mechanism of increased mtDNA copy number (76, 81). A pragmatic explanation is that increased mtDNA content provided cardiomyocytes with a kinetic reparative advantage after heart injury. However, the ubiquitous overexpression of hTFAM and Twinkle is associated with enlarged mtDNA nucleoids, and significant impairments to mtDNA replication and transcription (79, 80). Whether the overabundance of mtDNA nucleoids shifted mitochondrial ultrastructure and contributed to cardioprotection is also unclear (82). Although tissue-specific overexpression of mtDNA was beneficial, the physiological consequences of ubiquitous and constitutive mtDNA overexpression are also unclear, and few studies have examined the long-term impact of high mtDNA copy number *in vivo* (79). Work by Ylikallio et al. and colleagues suggests that hTFAM or Twinkle transgenic mice are plagued with age-graded increases in mito-dysfunction (79). Ylikallio et al. showed an aging-associated increase in mtDNA nucleoid enlargements and mtDNA deletions in the heart, brain, and skeletal tissues of hTFAM and Twinkle mice (79). The authors additionally reported mitochondrial cytochrome c oxidase deficiencies in the heart tissue of old hFTAM and Twinkle mice, indicative of decreased mitochondrial function (79). Contrasting work by Foote et al. has however reported that Twinkle transgenic mice have delayed cardiovascular aging (45). Compared to non-trangenic C57BL6-aged mice, Twinkle mice did not show mitochondrial deterioration (i.e., decreased mtDNA, decreased mitochondrial respiration, and increased ROS) in vascular tissue (45).

In essence, mtDNA quantity and quality wane with age, and studies suggest that preserving mtDNA quality delays aging on the cellular level. Understandably, transgenic models provide important but experimentally limited information regarding the translational potential of mtDNA repair, because of the unforeseen consequences of perpetual mtDNA upregulation. To this end, accumulating research suggests that mitochondrial transplantation can bridge this gap and serve as an anti-aging vascular therapy (83–89).

## Mitochondrial transplantation

Mitochondrial transplantation (mito-transfer) is a technique by which non-dysfunctional mitochondria are delivered to afflicted cells, tissues, or organs. Mito-transfer has also been reported to occur organically via direct cell-cell contact or extracellular vesicle transport (83–85). Proximal mito-transfer is facilitated by tunnel nanotubes (TNT), while distal mito-transfer is facilitated by microvesicles (83–85). Evidence of mito-transfer in cellular repair and dysfunctions has also been reported (83–87). For example, cancer cells can sequester mitochondria from T cells, likely contributing to T cell exhaustion and resistance to chemotherapy (87). Cardiomyocytes have been shown to dispose of dysfunctional mitochondria by vesicle budding (86). These vesicles are then scavenged by cardiac macrophages for degradation (86). The parameters that induce mito-transfer *in vivo* are unclear (83–85). Nevertheless, establishing whether *in vivo* intercellular mito-transfer is a fundamental process can be advantageous for delivering mitochondria to multiple cells from a single site.

Regarding CVD, mito-transfer has been used to ameliorate pathology in several animal models, particularly in heart failure (88, 89). Notability, Masuzawa et al. demonstrated that autologous mitochondria injected into ischemic hearts of rabbits confers cardio protection via enhanced ATP production, and upregulation of several cytokines associated with promoting angiogenesis (EGF, GRO, IL-6, MCP-3) (89). Importantly these improvements persisted for at least 28 days and mito-transfer of naked undamaged mitochondria did not induce cardiac or systemic inflammation (89). Separate work by Ikeda et al. demonstrated that the injection of mesenchymal stem cell derived mitochondria rich extracellular-microvescles (M-EVs) enhance the mitochondrial function of mouse cardiomyocytes after myocardial infarction (88). Mito-transfer will likely surpass the transgenic limitations of hTFAM and Twinkle mice, as related to issues of constitutive mtDNA overexpression. Further mechanistic insight is warranted, but current research suggests that mito-transfer can at minimum positively impact aging-associated mito-dysfunction in cardiovascular cells (88, 89).

Several factors must be accounted for before mito-transfer can be implemented in a clinical setting to treat cardiovascular aging. First, the inherent variability of mtDNA heteroplasmy and aging-associated mtDNA damage in humans may complicate defining a standard mito-transfer dose. Studies also show that mtDNA exists as different haplotypes, with each haplotype displaying different ETC expressions (90). The consequences of mtDNA haplotype mixing are unclear (90). Theoretically, the expression of a different mtDNA haplotype is only troubling if said haplotype promotes mitochondrial dysfunction (91). Second, The ethical isolation and harvesting of mitochondria are also important factors to consider. Autologous mitochondria can be isolated from lab grown IPSCs (92). However, current literature suggests that mitochondrial dysfunctions can manifest after IPSC differentiation in an age-dependent manner (93).

Lastly, the least immunogenic methods for extracellular mito-transfer and mtDNA delivery are still unresolved (94–97). Internal mitochondrial components that become extracellularly exposed serve as damage associated molecular patterns (DAMPS/mitoDAMPS), that initiate and amplify pro-inflammatory signaling cascades (70, 71, 98). Work by Chang et al. demonstrated that functional allogeneic mitochondria can be intranasally delivered to the brain of 6-hydroxydopamine (6-OHDA)-lesioned rats (99). Although the mito-transfer improved mitochondrial function and dopaminergic neuron viability, intranasal delivery increased the expression of several pro-inflammatory cytokines, including IL-1α, IL-1β, and IL-17A (99). Interestingly, Dache et al. reported on the presence of intact, fully functional non encased (i.e., cell free) mitochondria in the plasma of healthy individuals (100). These findings are thought provoking, as they suggest mitochondria encapsulation may not be required for systemic health benefits. Another inference from Dache et al.'s work is that systemic inflammation associated with mito-transfer may be related to healthy mitochondrial infusions being contaminated with damaged mitochondria. In this regard, work by Zhang et al. (101) demonstrated that systemic injection of autologous mitochondria isolated from the skeletal muscle of mice, reduced systemic inflammation (IL-6 and IL-1β) and enhanced bacterial clearance in an acute model of sepsis (101).

In conclusion, both the magnitude of dysfunctional mitochondria and damaged mtDNA are higher in the elderly. Since less damaged mtDNA is present in younger cells, these mtDNA heteroplasmy profiles more readily support the synthesis of non-dysfunctional mtDNA derived proteins. Current literature supports the further exploration of mito-transfer as an anti-aging therapy in the cardiovascular compartment. Several limitations must also be addressed prior to implementation of mito-transfer as therapy. Whether, mito-transfer confers the same magnitude of benefits to young cells, as what is to be anticipated in aged cells, is also an intriguing thought.
